# Clemastine Fumarate Protects Against Myocardial Ischemia Reperfusion Injury by Activating the TLR4/PI3K/Akt Signaling Pathway

**DOI:** 10.3389/fphar.2020.00028

**Published:** 2020-02-10

**Authors:** Xiaoxiao Yuan, Zhaodong Juan, Rui Zhang, Xiaotong Sun, Ru Yan, Feng Yue, Yaru Huang, Jiacheng Yu, Xiaohui Xia

**Affiliations:** Shandong Provincial Medicine and Health Key Laboratory of Clinical Anesthesia, Department of Anesthesiology, Weifang Medical University, Weifang, China

**Keywords:** myocardial ischemia-reperfusion injury, inflammatory response, apoptosis, TLR4/PI3K/Akt, clemastine fumarate

## Abstract

Our pilot studies have shown that clemastine fumarate (CLE) can protect against myocardial ischemia-reperfusion injury (MIRI) through regulation of toll like receptor 4 (TLR4). However, the protective mechanism of CLE and related signaling pathways for MIRI remains unclear. The objective of this study is to determine the mechanism by which CLE relieves MIRI in cardiomyocytes and its relationship with the TLR4/PI3K/Akt signaling pathway. CCK8 analysis was used to test the optimal concentration of TLR4 inhibitor CLI-095 and TLR4 agonist lipopolysaccharide (LPS) on MIRI. The expression of inflammatory factors, oxidative stress response, cell damage, and intracellular calcium redistribution of cardiomyocytes were examined using the ELISA kits, Total Superoxide Dismutase Assay Kit with WST-8 and Lipid Peroxidation MDA Assay Kit, LDH Cytotoxicity Assay Kit, and laser scanning confocal microscope. The expression of TLR4/PI3K/Akt and cleaved caspase-3 were determined by Western blotting and immunofluorescent staining. Our results showed that MIRI aggravated the inflammatory response, oxidative stress, cellular damage of cardiomyocytes, and caused redistribution of intracellular calcium, upregulated the expression of TLR4 protein, cleaved caspase-3 protein, and down-regulated the expression of PI3K/Akt protein. After treatment with CLE, the inflammatory response, oxidative stress, and cellular damage of cardiomyocytes were alleviated, and intracellular calcium ion accumulation decreased. The expression of TLR4 protein, cleaved caspase-3 protein declined, but PI3K/Akt protein expression increased in cardiomyocytes treated with CLE. In addition, after treatment with the TLR4 inhibitor CLI-095, the results were similar to those of CLE treatment. The TLR4 agonist LPS aggravated the reactions caused by MIRI. The role of LPS was reversed after CLE treatment. These results suggested that CLE can attenuate MIRI by activating the TLR4/PI3K/Akt signaling pathway.

## Introduction

Timely myocardial reperfusion using either thrombolytic therapy or primary percutaneous coronary intervention (PPCI) is the effective treatment to limit the size of myocardial infarct (MI), preserve left-ventricular systolic function, and reduce the onset of heart failure as well as morbidity and mortality of patients with ischemic cardiomyopathy. Therefore, there is increasing interest in studying myocardial ischemia-reperfusion injury (MIRI) in recent years. However, MIRI is considered a common complication that occurs during reperfusion treatment (Liu et al., 2016).

MIRI refers to the pathological process of progressive myocardial injury, and the tissue damage is progressively aggravated when the coronary artery is partially re-open after a certain period of time (Wei et al., 2014). Myocardial ischemia-reperfusion can cause severe arrhythmias, leading to sudden death (Frank et al., 2012; Gerczuk and Kloner, 2012; Hausenloy and Yellon, 2013). The main mechanism of MIRI is related to the massive release of free oxygen radicals, loss of intracellular and mitochondrial calcium homeostasis, an inflammatory cascade, promotion of apoptosis, and myocardial necrosis (Vincent et al., 2017).

MIRI treatment is very important for the prevention of myocardial infarction. However, there is no effective advanced treatment plan available (Wang et al., 2015). Apoptosis is the main mechanism of death in MIRI cardiomyocytes. Therefore, inhibition of inflammation and apoptosis is a useful therapeutic direction for MIRI. Furthermore, in order to inhibit apoptosis at an earlier stage, research should be directed to the upstream of the apoptotic pathway (Xia et al., 2016).

The phosphatidylinositol 3-kinase (PI3K)/protein kinase B (Akt) signaling pathway is one of the most critical signaling pathways regulating cell survival and plays an important role in myocardial protection in MIRI (Zhang et al., 2010; Zhang et al., 2014; Thokala et al., 2017). Akt is a downstream target of PI3K; when Akt is activated by upstream signaling factor PI3K-phosphoinositide-dependent kinase-1, it activates glycogen synthase kinase (GSK)-3β to phosphorylated (p)-GSK-3β, which is believed to be the integrated point of several pathways and has an important role in cardioprotection (Juhaszova et al., 2009). Cysteine aspartate protease-3 (caspase-3) is one of the most important enzymes in the caspase family, and is involved in the apoptotic process following activation by other members of the caspase family. Caspase-3 is the “hallmark enzyme” of cell apoptosis (Zhou et al., 2015). Cleaved caspase-3 is the activated form of caspase-3. Therefore, when Akt is activated, MIRI-induced myocardial injury may be reduced *via* the involvement of GSK-3β and caspase-3 (Cheng et al., 2016). Li et al. (2016) found that short-term pretreatment with hesperidin could reduce inflammatory response and oxidative stress through the activation of the PI3K/Akt pathway and HMGB1, reduce myocardial cell apoptosis in MIRI, and play a protective role in myocardial protection. Our previous study also confirmed that the protective effect of *Acanthopanax senticosus* and pioglitazone on ischemia-reperfusion cardiomyocytes was related to the PI3K/Akt signaling pathway. However, studies on the PI3K/Akt pathway has not achieved satisfactory results when applied in clinical trials (Li and Wang, 2014), and possible prevention measures based on the PI3K/Akt pathway mechanism have not yet been studied. Previous studies have found that toll-like receptor 4 (TLR4) is an upstream factor of the PI3K/Akt signaling pathway (Ha et al., 2011). Therefore, delineating the TLR4/PI3K/Akt signaling pathway could be helpful in the treatment of MIRI.

TLR4 is one of the pattern recognition receptors. It plays a crucial role in the induction of inflammatory response and activates downstream signaling in myocardial ischemia (Akira et al., 2001; Baumgarten et al., 2001; van der Kaaij and Bogers, 2008). Studies have found that TLR4 plays an important regulatory role in MIRI. Blocking TLR4 signaling pathway can reduce the inflammatory response of MIRI and reduce myocardial cell apoptosis (Yang et al., 2015; Zhang et al., 2017). Clemastine fumarate (CLE) belongs to the second generation of H1 receptor blockers and is clinically used to treat various allergic diseases induced by histamine. Studies have shown that CLE not only has antihistamine effect, but also inhibits NF-κB activity (Leurs et al., 2002) and down-regulates TLR4 expression (Eppinger et al., 1995), reducing the synthesis of pro-inflammatory factors and exerting anti-inflammatory effects. Our previous study indicated that CLE could inhibit the expression of TLR4 in myocardial tissue, reduce myocardial ischemia-reperfusion injury and plays a role in myocardial protection. The present study is designed to investigate whether CLE exerts cardio-protective effects during MIRI in cardiomyocytes through the TLR4/PI3K/Akt signaling pathway.

## Materials and Methods

### Antibodies and Reagents

CLE was obtained from Huarun Shuanghe Limin Pharmaceutical Co., Ltd. (2 ml:2 mg; Jinan, China). TLR4 antibody was purchased from Affinity Biologicals (Cincinnati, USA). TLR4 Inhibitor, CLI-095, was purchased from Invitrogen (product purity 99.95%; California, USA). TLR4 agonist lipopolysaccharide (LPS) was received from Sigma (Product purity ≥ 99%; MO, USA). DMEM/HIGH GLUCOS Medium and DMEM/LOW GLUCOSE Medium were purchased from HyClone (Utah, USA). Fetal bovine serum (FBS) was obtained from Gibco (New York, USA). Newborn bovine serum (NBS) was obtained from Every Green (Taiwan, China). Fetal equine serum (HS) and 5-Bromo-2-deoxyUridine (Brdu) were obtained from Solarbio (Beijing, China). Tumor necrosis factor (TNF)-α and interleukin (IL)-1β enzyme-linked immunosorbent assay (ELISA) kits were purchased from Dakowei Biotechnology Co., Ltd (Beijing, China). LDH Cytotoxicity Assay Kit, Total Superoxide Dismutase Assay Kit with WST-8, Lipid Peroxidation MDA Assay Kit, and Fluo-4 AM were all obtained from Beyotime Biotechnology (Shanghai, China). Hiscript II Q RT SuperMix for Qpcr (+gDNA wiper) and ChamQ SYBR qPCR Master Mix were purchased from Vazyme (Nanjing, China). All reagents and antibodies were of analytical grade and commercially available.

### Cell Culture

The cardiomyocyte H9C2 cells and HL-1 cells were purchased from Procell Life Science & Technology Co., Ltd (Wuhan, China). H9C2 cells and HL-1 cells were plated onto 25 cm^2^ culture flasks at a density of 1 × 10^6^ cells/ml in Dulbecco’s modified Eagle’s medium (DMEM) containing 10% fetal bovine serum, 100 units/ml penicillin/streptomycin in an incubator with 5%CO_2_ at 37°C. H9C2 cells and HL-1 cells were randomly assigned to six groups: 1) control group (CON), cells were maintained in an environment with 5% CO_2_ at 37°C; 2) MIRI group (MIRI), cells received 4 h of hypoxia and 4 h of reoxygenation; 3) CLE group (MIRI+CLE), cells received 4 h of hypoxia, and 4 h of reoxygenation, in which 1.25 μg/ml CLE was added to the cells during reoxygenation; 4) TLR4 inhibitor CLI-095 group (MIRI+095), cells received 4 h of hypoxia, and 4 h of reoxygenation, in which 1.0 μg/ml CLI-095 was added to the cells during reoxygenation; 5) TLR4 agonist LPS group (MIRI+LPS), cells received 4 h of hypoxia, and 4 h of reoxygenation, in which 5.0 μg/ml LPS was added to the cells during reoxygenation; 6) LPS and clemastine fumarate group (MIRI+LPS+CLE), cells received 4 h of hypoxia, and 4 h of reoxygenation, in which suitable concentrations CLE and LPS was added to the cells during reoxygenation. Primary cardiacmyocytes (CMs) was isolated from the hearts of healthy SD rats aged 1–3 days for primary culture. Briefly, 1–3 day-old rats were sterilized twice with 75% alcohol. Ventricles were separated from neonatal rats, minced and washed in cold liquid (10 Mm HEPES, 0.4%HS, D-Hanks). The heart tissue was dissociated using a digestion solution [200 U/ml collagenase II, 1% P/S (100 U/ml penicillin and 0.1 g/l streptomycin)] in an Erlenmeyer flask containing rotor. Then, the flask was placed in an oscillator at 37°C. We pooled cell suspensions and centrifuged the mixture 700 rpm for 5 min. We then resuspended the cells in DMEM/high glucose supplemented with 5% NBS and 8% HS and 1% P/S. After that, we filtered these cell suspensions with cell strainer and plated the cells onto 25 cm^2^ cell culture flasks. The cells were plated for 60 min to allow CFs to preferentially attach to the bottom of the culture dishes. The collected CMs were directly plated at a density of 1x10^5^ cells/cm ² onto cell culture flasks and cultured in DMEM/high glucose supplemented with 5% NBS, 8% HS, 1% P/S, and 0.1 mM Brdu. Cells were then plated at 37°C in an incubator with 5% CO_2_. Culture media of CMs was changed every 2 days. The grouping of CMs was the same as mentioned above.

### Establishment of a Myocardial Ischemia-Reperfusion Injury Model in H9C2 Cells, HL-1 Cells, and Cardiacmyocytes

To mimic the *in vivo* MIRI model, H9C2 cells, HL-1 cells, and CMs at ~90% confluence were incubated with low-sugar DMEM without FBS for 2 h in an environment with 5% CO_2_ at 37°C. Then, the cells were transferred to a hypoxic environment (37°C, 5% CO_2_, 95% N_2_) for 4 h. The H9C2 cells and HL-1 cells were provided with high glucose DMEM containing 10% FBS. CMs were provided with high glucose DMEM containing 5% NBS, 8% HS, 1% P/S, and 0.1 mM Brdu. Then, they were moved to a CO_2_ Incubator (37°C, 5% CO_2_) for reoxygenation. The cells were harvested 4 h post-reoxygenation for further analyses.

### Cell Viability Assay on Cells Treated With Different Doses of CLI-095 and Lipopolysaccharide

The viability of cells after treatment with different doses of CLI-095 and LPS were assessed using the Cell Counting Kit-8 (Beijing Solarbio Science & Technology Co., Ltd). The cells were cultured in a 96-well plate at a density of 1 × 10^4^ cells/well and incubated for 24 to 48 h. When the cells were well-grown, an ischemia-reperfusion model was made to study the protective mechanism of CLE on MIRI. In the MIRI+095 group, CLI-095 was added at final concentrations of 0.008, 0.04, 0.2, 1, 5, 25, and 50 μg/ml; LPS was added to the MIRI+LPS group at final concentrations of 0.008, 0.04, 0.2, 1, 5, 25, and 50 μg/ml. After 4 h, the original medium was discarded and 100 μl of medium containing CCK-8 at a concentration of 10 mg/ml was added in each well and incubated with an additional 4 h. The optical density (OD) values at 450 nm were measured using a microplate reader. Each experiment was repeated three times.

### Measurement of Lactate Dehydrogenase Production in Culture Medium

The cells were treated under different conditions, and the cell culture supernatant were collected from each group. Then, the lactate dehydrogenase (LDH) enzyme activity (mU/ml) in culture supernatant of different samples was measured by the LDH Cytotoxicity Assay Kit according to the manufacturer’s instructions.

### Observation of Intracellular Calcium Ion Distribution by Laser Scanning Confocal Microscopy

The H9C2 cells were seeded in a laser confocal culture dish. The cells were incubated with 1-[2-amino-5-(2,7-difluoro-6-hydroxy-3-oxo-9-xanthenyl)phenoxy]-2-(2-amino-5-methylphenoxy)ethane-N,N,N’,N’-tetraaceticacid,pentaacetoxymethyl ester (Fluo-4 AM) for 30 min in the dark at 37°C. After washing three times with phosphate-buffered saline (PBS), the cells were incubated with Fluo-4 AM one more time using the same conditions. Finally, the redistribution state of intracellular calcium ions was observed under a laser scanning confocal microscope.

### Enzyme-Linked Immunosorbent Assay Analysis for Tumor Necrosis Factor-α and Interleukin-1β Concentration in Cell Culture Supernatant

The cells were treated under different conditions, and different groups of cell culture supernatant was collected. Then, the level of inflammatory cytokines TNF-α and IL-1β in culture supernatant were measured by ELISA kits according to the manufacturer’s instructions. The optical density (OD) values at 450 nm were measured using a microplate reader.

### Determination of Oxidative Parameters

The proteins of cells from different groups were extracted. After quantitation of protein concentration using BCA Protein Assay Kit, superoxide dismutase (SOD) activity (U/mg), and malondialdehyde (MDA) concentration (μmol/μg protein) were determined using Total Superoxide Dismutase Assay Kit with WST-8 and Lipid Peroxidation MDA Assay Kit according to the manufacturer’s instructions.

### Detection of Tumor Necrosis Factor-α and Interleukin-1β Messenger Ribonucleic Acid Expression in Cardiomyocytes and HL-1 Cells

Real-time PCR was used to detect the messenger RNA (mRNA) expressions of TNF-α and IL-1β in CMs and HL-1 cells. Total RNA was isolated from CMs and HL-1 cells with TRIzol Reagent (Thermo Fisher Scientific, Shanghai, China), and converted to complementary DNA (cDNA) using the Hiscript II Q RT SuperMix for Qpcr (+gDNA wiper) (Vazyme, Nanjing, China). Real-time PCR was performed using ChamQ SYBR qPCR Master Mix (Vazyme, Nanjing, China) with a LightCycler 480 II (Roche, Indianapolis, IN, USA). Briefly, 10 μl PCR master mix was prepared as follows: 4.8 μl cDNA template (15 ng/μl), 5 μl 2×ChamQ SYBR qPCR Master Mix, and 0.2 μl gene-specific primers (10 μM). The PCR amplification protocol was the following: 5 min at 95°C (stage 1:predegeneration), 40 cycles at 95°C for 10 s, and 60°C for 30 s (stage 2:circular reaction). The expression levels of target genes were normalized to the average levels of glyceraldehyde 3-phosphate dehydrogenase (GAPDH). The specificity of PCR primers (**Tables 1** and **2**) were confirmed with 1% agarose gel electrophoresis and melt curves. The fold difference for the mRNA expression level was calculated using 2^−ΔΔCt^.

**Table 1:** Primers used for real-time (RT)-PCR analysis of tumor necrosis factor (TNF)-α and IL-1β messenger RNA (mRNA) in cardiacmyocytes (CMs).

Primer name	Sequence
TNF-α forward	TGATCGGTCCCAACAAGGA
TNF-α reverse	TGCTTGGTGGTTTGCTACGA
IL-1β forward	GGGATGATGACGACCTGC
IL-1β reverse	CCACTTGTTGGCTTATGTT
GAPDH forward	GTTACCAGGGCTGCCTTCTC
GAPDH reverse	ACCAGCTTCCCATTCTCAGC

**Table 2:** Primers used for real-time (RT)-PCR analysis of tumor necrosis factor (TNF)-α and interleukin (IL)-1β messenger RNA (mRNA) in HL-1 cells.

Primer name	Sequence
TNF-α forward	TGCTCTGTGAAGGGAATGGG
TNF-α reverse	ACCCTGAGCCATAATCCCCT
IL-1β forward	ATGAAAGACGGCACACCCAC
IL-1β reverse	AAGGCAGAGTCTTCGGTGAG
GAPDH forward	CCCTTAAGAGGGATGCTGCC
GAPDH reverse	ACTGTGCCGTTGAATTTGCC

### Western Blot Analyses

Cultured cells were lysed in Lysis Buffer (Beijing Solarbio Science & Technology Co., Ltd.) on ice for 30 min, and the lysates were clarified by centrifugation at 4°C for 5 min at 12.0×RPM. After quantitation of protein concentration using BCA Protein Assay Kit (CWBIO, Beijing, China), total protein was heated at 100°C for 5 min. Each sample contained 30 μg of protein was used for electrophoresis and separated by 10% sodium dodecyl sulfate polyacrylamide gel electrophoresis (SDS-PAGE) and then transferred to nitrocellulose membranes (Millipore, MA). The membranes were blocked for 2 h at room temperature with 5% non-fat dry milk, then incubated overnight at 4°C with primary antibodies including TLR4 (1:1,000 dilution, AF7017; Affinity), PI3K (0.05 μg/ml, Cat.#09-482; Millipore), p-Akt (1:7,000 dilution, ab81283; Abcam), Akt (0.5 g/ml, Cat.#05-591; Millipore), caspase3 (1:1,000 dilution, #9662; Cell Signal Technology), and cleaved caspase-3 (1:1,000 dilution, #9661; Cell Signal Technology). GAPDH (Zsbio Commerce, Beijing, China) or β-actin (Beyotime Biotechnology, Jiangsu, China) was used as an internal reference. After washing with Tris buffered saline with Tween 20 (TBST) three times, goat anti-rabbit immunoglobulin G (IgG) (H+L) horseradish peroxidase (HRP) (1:5,000 dilution, GAR007; MultiSciences) or goat anti-mouse IgG (H+L) HRP (1:5,000 dilution, GAM007; MultiSciences) was incubated with the membrane for 2 h at room temperature and then washed three times with TBST. Bands were visualized with enhanced chemiluminescence (ECL) detection reagents (CWBIO, Beijing, China) using an ECL assay. The relative band intensity was measured by Image-Pro Plus software.

### Immunofluorescent Staining

Cells were washed three times for 5 min each with PBS, fixed for 30 min in 4% paraformaldehyde, washed three times for 5 min each in PBS. Next, 5% normal goat blocking serum (Solarbio Science & Technology Co., Ltd, Beijing, China) was used to block cells for 30 min at 37°C. The cells were then incubated with primary antibody overnight at 4°C using TLR4 (1:200 dilution, AF7017; Affinity), PI3K (1:5,000 dilution, #09-48; Millipore), p-Akt (1:200 dilution, ab81283; Abcam), and cleaved caspase-3 (1:400 dilution, #9661; Cell Signal Technology). After being washed three times for 10 min each with PBS, the cells were incubated with goat anti-rabbit IgG (H+L), fluorescein isothiocyanate (FITC) (1:500 dilution, GAR001; MultiSciences), or goat anti-mouse IgG (H+L), FITC (1:500 dilution, GAM001; MultiSciences) for 40 min in the dark at 37°C. The cells were again washed three times with PBS and treated for 5 min at 37°C with 4’,6-diamidino-2-phenylindole (DAPI) (1:200 dilution, C1005; Beyotime) to stain the cell nuclei. Finally, after being washed three times with PBS, the cells were sealed with Antifade Mounting Medium (P0126; Beyotime) and observed using a fluorescence microscope.

### Statistical Analysis

All data were processed using SPSS 20.0 statistical software, and the measurement data were expressed as the mean ± standard deviation (SD). Comparison of multiple groups was analyzed by one-way analysis of variance (ANOVA), and pairwise comparison of the study was detected by t test. The results were considered statistically significant if *P* < 0.05.

## Results

### Effects of CLI-095 and Lipopolysaccharide on the Viability of H9C2 Cells

**Figure 1:**
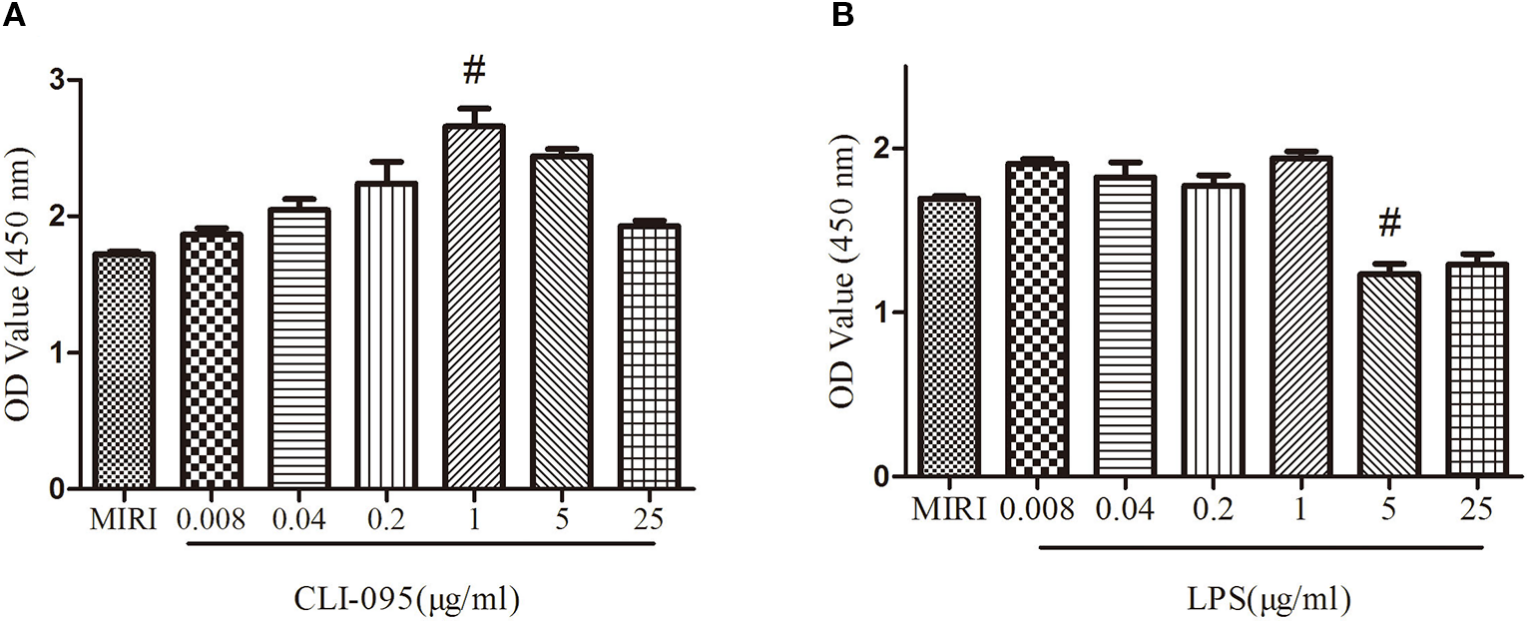
Comparison of different doses of CLI-095 and lipopolysaccharide (LPS) on H9C2 cardiomyocytes viability using CCK-8 assay. **(A)** Statistical analysis on cardiomyocytes viability using different doses of CLI-095. **(B)** Statistical analysis graph showing cardiomyocytes viability using different doses of LPS. ^#^indicates a significant difference compared with myocardial ischemia-reperfusion injury (MIRI) group, *P* < 0.05.

The viability of the cardiomyocytes treated with different doses of CLI-095 and LPS was examined using the CCK-8 assay. As depicted in **Figure 1A**, the cell viability increased with the treatment of CLI-095 at the concentration of 1 μg/ml, compared with MIRI group (*P* < 0.05). The survival rate of cardiomyocytes decreased when cells were treated with LPS at 5 μg/ml (**Figure 1B**) (*P <* 0.05).

### Effects of Clemastine Fumarate, CLI-095, and Lipopolysaccharide Treatment on the Injury of H9C2 Cells

**Figure 2:**
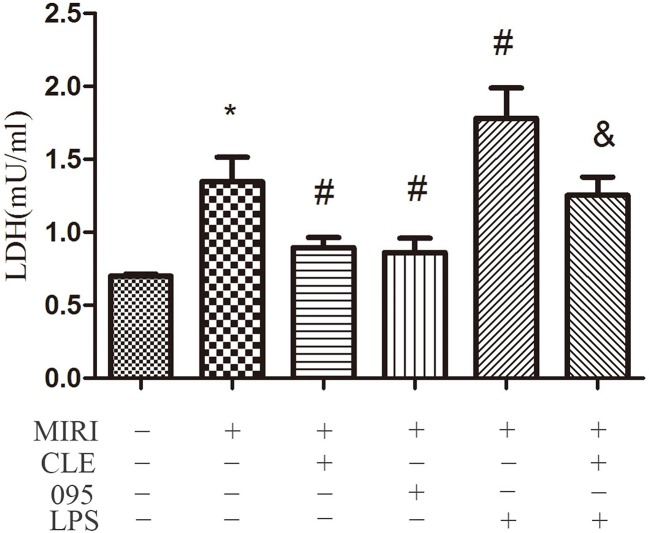
Comparison of release of lactate dehydrogenase (LDH) in cardiomyocytes culture medium by LDH Cytotoxicity Assay Kit. Statistical analysis graph of each group indicating the release of LDH in cardiomyocytes culture fluid. Error bars represent SD (standard deviation), *indicates a significant difference compared with CON group, *P* < 0.05; ^#^indicates a significant difference compared with myocardial ischemia-reperfusion injury (MIRI) group, *P* < 0.05; ^&^indicates a significant difference compared with MIRI+lipopolysaccharide (LPS) group, *P* < 0.05.

To assess the effect of CLE on MIRI injury in cardiomyocytes and the role of TLR4 in this response, LDH levels in supernatant of H9C2 cardiomyocytes were determined using the LDH Cytotoxicity Assay Kit. As shown in **Figure 2**, the level of LDH in the MIRI group was significantly higher than that in the CON group compared with the MIRI group. The LDH levels in the MIRI+CLE group and the MIRI+095 group were significantly lower, and the LDH level in the MIRI+LPS group was significantly higher. Compared with the MIRI+LPS group, the LDH level in the MIRI+LPS+CLE group was significantly lower (*P* < 0.05). These results suggested that CLE can attenuate myocardial damage caused by myocardial ischemia-reperfusion injury. The TLR4 inhibitor CLI-095 attenuated myocardial damage caused by MIRI similar to CLE. The TLR4 agonist LPS aggravated myocardial damage. These results suggested that the use of CLE can alleviate the effect of LPS.

### Effects of Clemastine Fumarate, CLI-095, and Lipopolysaccharide Treatment on the Intracellular Calcium Ion Distribution in MIRI H9C2 Cells

**Figure 3:**
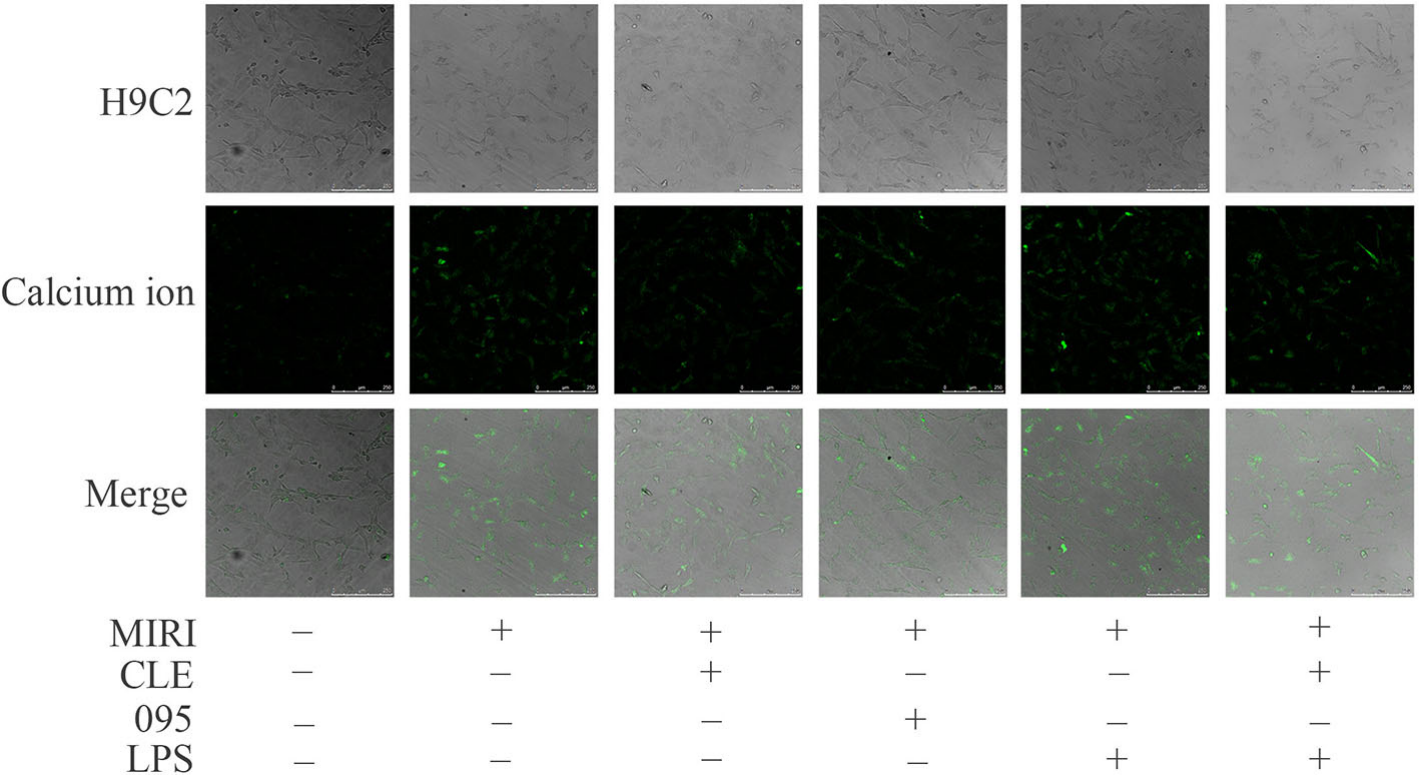
Comparison of intracellular calcium ion distribution in myocardial ischemia-reperfusion injury (MIRI) cardiomyocytes using laser scanning confocal microscopy. Laser scanning confocal microscopy showing intracellular calcium ion distribution in H9C2 cardiomyocytes of each group. Green represents calcium ion fluorescence. Scale bar: 50 μm.

Myocardial ischemia-reperfusion injury causes dysregulation of calcium, which eventually leads to calcium overload in cells. It in turn stimulates a series of pathological changes and even cell death. To assess the effect of CLE on intracellular calcium ion distribution during MIRI and the role of TLR4 in this reaction, laser scanning confocal microscopy was used to observe the calcium ion fluorescence-positive reaction. As shown in **Figure 3**, compared with the control group, the fluorescence intensity of intracellular calcium ions in the MIRI group was significantly enhanced; compared with the MIRI group, the fluorescence intensity of intracellular calcium ions in the MIRI+CLE group and the MIRI+095 group was significantly weakened, and the intensity in the MIRI+LPS group was significantly enhanced; compared with the MIRI+LPS group, the fluorescence intensity of the MIRI+LPS+CLE group was significantly weakened. This result suggested that CLE can attenuate calcium ion redistribution caused by myocardial ischemia-reperfusion injury. Using the TLR4 inhibitor CLI-095, the results showed that the intracellular calcium ion distribution was reduced as with CLE; while the TLR4 agonist LPS increased the intracellular calcium ion distribution. These results indicated that the use of CLE alleviated the effect of LPS.

### Clemastine Fumarate, CLI-095, and Lipopolysaccharide Treatment on the Release of Tumor Necrosis Factor-α and Interleukin-1β in Myocardial Ischemia-Reperfusion Injury H9C2 Cells and the Messenger Ribonucleic Acid Expression of Tumor Necrosis Factor-α and Interleukin-1β in Myocardial Ischemia-Reperfusion Injury Cardiacmyocytes and HL-1 Cells

**Figure 4:**
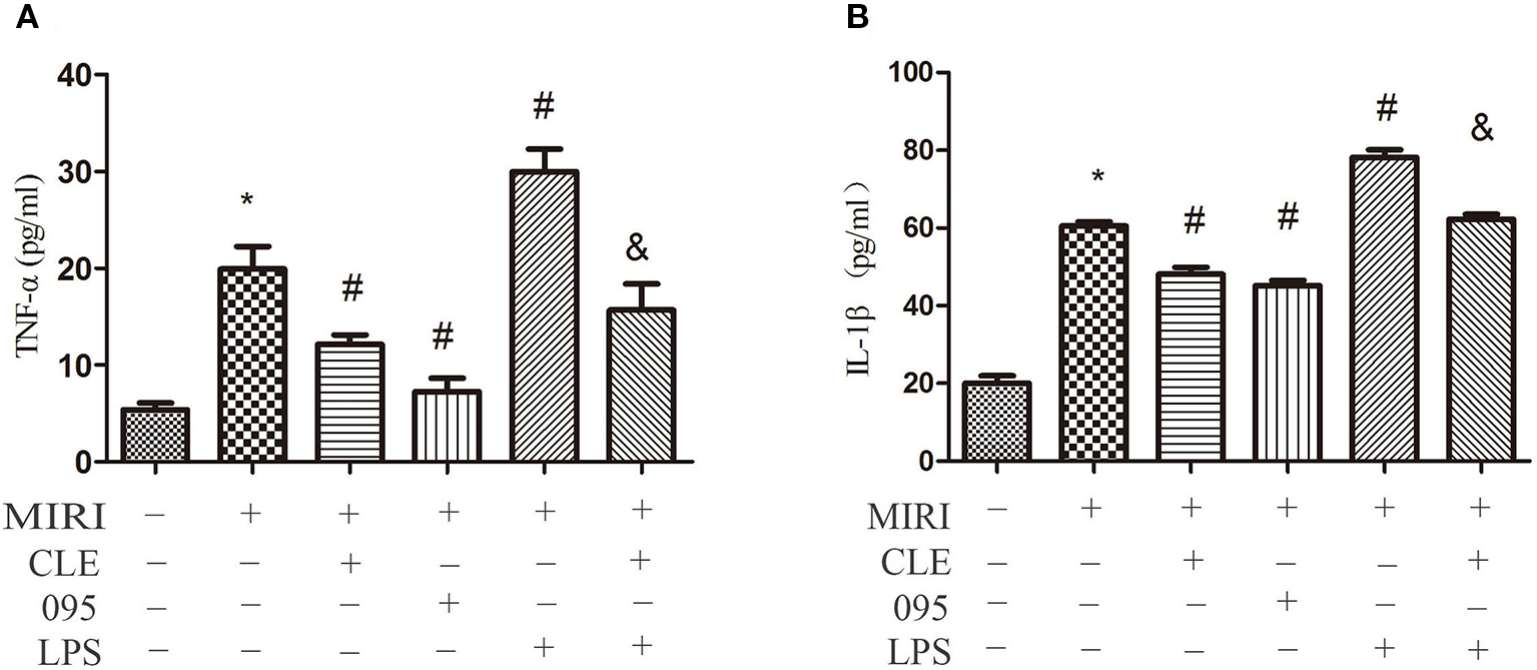
Comparison of release of tumor necrosis factor (TNF)-α and interleukin (IL)-1β in cardiomyocyte culture medium by ELISA kits. **(A)** Statistical analysis graph of each group indicating the release of TNF-α in cardiomyocytes culture fluid. **(B)** Statistical analysis graph of each group indicating the release of IL-1β in cardiomyocytes serum. Error bars represent SD (standard deviation), *indicates a significant difference compared with CON group, *P* < 0.05; ^#^indicates a significant difference compared with myocardial ischemia-reperfusion injury (MIRI) group, *P* < 0.05; ^&^indicates a significant difference compared with MIRI+LPS group, *P* < 0.05.

The release of inflammatory factors, such as TNF-α, IL-1β, and others, have been found to play important roles in mediating and exacerbating myocardial ischemia/reperfusion injury. To evaluate the effect of CLE on the inflammatory response of MIRI cardiomyocytes and the role of TLR4 in this response, the release of TNF-α and IL-1β in H9C2 cardiomyocytes culture medium were determined by ELISA kits. In addition, in order to verify the effect of CLE on MIRI CMs and HL-1 cells, we tested the TNF-α and IL-1β expression at mRNA levels by real-time PCR the mRNA expression of TNF-α and IL-1β in CMs and HL-1 cells were determined by Real-time PCR. The results in **Figures 4A, B** showed that levels of TNF-α and IL-1β in the cell culture media of H9C2 cells in the MIRI group were significantly elevated in contrast to the control group. Compared with the MIRI group, TNF-α and IL-1β levels were significantly lower in the MIRI+CLE group and the MIRI+095 group, and significantly increased in the MIRI+LPS group. In addition, the levels of TNF-α and IL-1β in the MIRI+LPS+CLE group were significantly lower than those in the MIRI+LPS group (both *P* < 0.05). The results in **Supplemental Figures 1**, **2** showed the mRNA expression of TNF-α and IL-1β in CMs and HL-1 cells, respectively. The MIRI group was significantly elevated in contrast to the control group. Compared with the MIRI group, TNF-α and IL-1β mRNA expression levels were significantly lower in the MIRI+CLE group and the MIRI+095 group, and significantly increased in the MIRI+LPS group. In addition, the mRNA expression levels of TNF-α and IL-1β in the MIRI+LPS+CLE group were significantly lower than those in the MIRI+LPS group (both *P* < 0.05). This result suggested that CLE can inhibit inflammatory response caused by MIRI in H9C2 cells, CMs, and HL-1 cells. The TLR4 inhibitor CLI-095 had the same effect, while TLR4 agonist LPS had an opposite effect and CLE attenuated the effect of LPS.

### Effects of Clemastine Fumarate, CLI-095, and Lipopolysaccharide Treatment on Oxidative Response in Myocardial Ischemia-Reperfusion Injury H9C2 Cells

**Figure 5:**
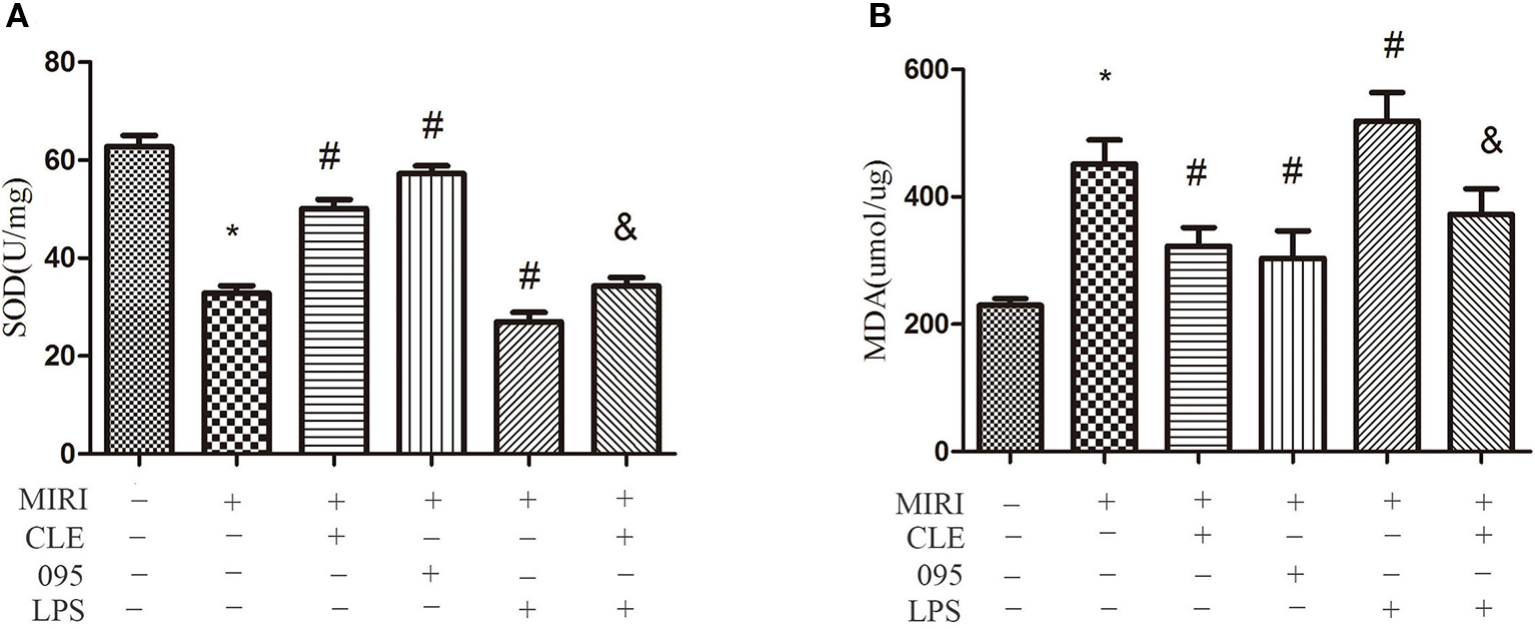
Comparison of SOD vitality by Total Superoxide Dismutase Assay Kit with WST-8 and MDA content by Lipid Peroxidation MDA Assay Kit in MIRI H9C2 cardiomyocyte. **(A)** Statistical analysis graph of each group indicating SOD vitality. **(B)** Statistical analysis graph of each group indicating MDA content. Error bars represent SD (standard deviation), *indicates a significant difference compared with CON group, *P* < 0.05; ^#^indicates a significant difference compared with myocardial ischemia-reperfusion injury (MIRI) group, *P* < 0.05; ^&^indicates a significant difference compared with MIRI+lipopolysaccharide (LPS) group*, P* < 0.05.

Oxidative stress reaction occurs when myocardial ischemia-reperfusion injury happens. To assess the effect of CLE on oxidative stress of MIRI cardiomyocytes and the role of TLR4 in this response, SOD and malondialdehyde (MDA) content in H9C2 cardiomyocytes were detected using the Total Superoxide Dismutase Assay Kit with WST-8 and Lipid Peroxidation MDA Assay Kit. As depicted in **Figure 5A**, SOD activity in the MIRI group was significantly lower than that in the control group. Compared with MIRI group, SOD activity in the MIRI+CLE group and the MIRI+095 group was significantly enhanced, and that in the MIRI+LPS group was significantly weakened; compared with the MIRI+LPS group, SOD activity in the MIRI+LPS+CLE group was significantly enhanced (both *P* < 0.05). **Figure 5B** showed that the MDA content in the MIRI group was significantly higher than that in the control group; compared with the MIRI group, MDA content in the MIRI+CLE group and the MIRI+095 group was significantly lower than that in the MIRI+LPS group; compared with the MIRI+LPS group, MDA content in the MIRI+LPS+CLE group was significantly lower (both *P* < 0.05). These results suggested that CLE exerts an antioxidant effect on MIRI cardiomyocytes. The use of the TLR4 inhibitor CLI-095 can achieve the same effect as CLE; while the TLR4 agonist LPS can aggravate oxidative stress, and CLE can reverse the action of LPS.

### Effects of Clemastine Fumarate, CLI-095, and Lipopolysaccharide Treatment on TLR4, PI3K, P-Akt/Akt Protein Levels in Myocardial Ischemia-Reperfusion Injury H9C2 Cells, Cardiacmyocytes, and HL-1 Cells

**Figure 6:**
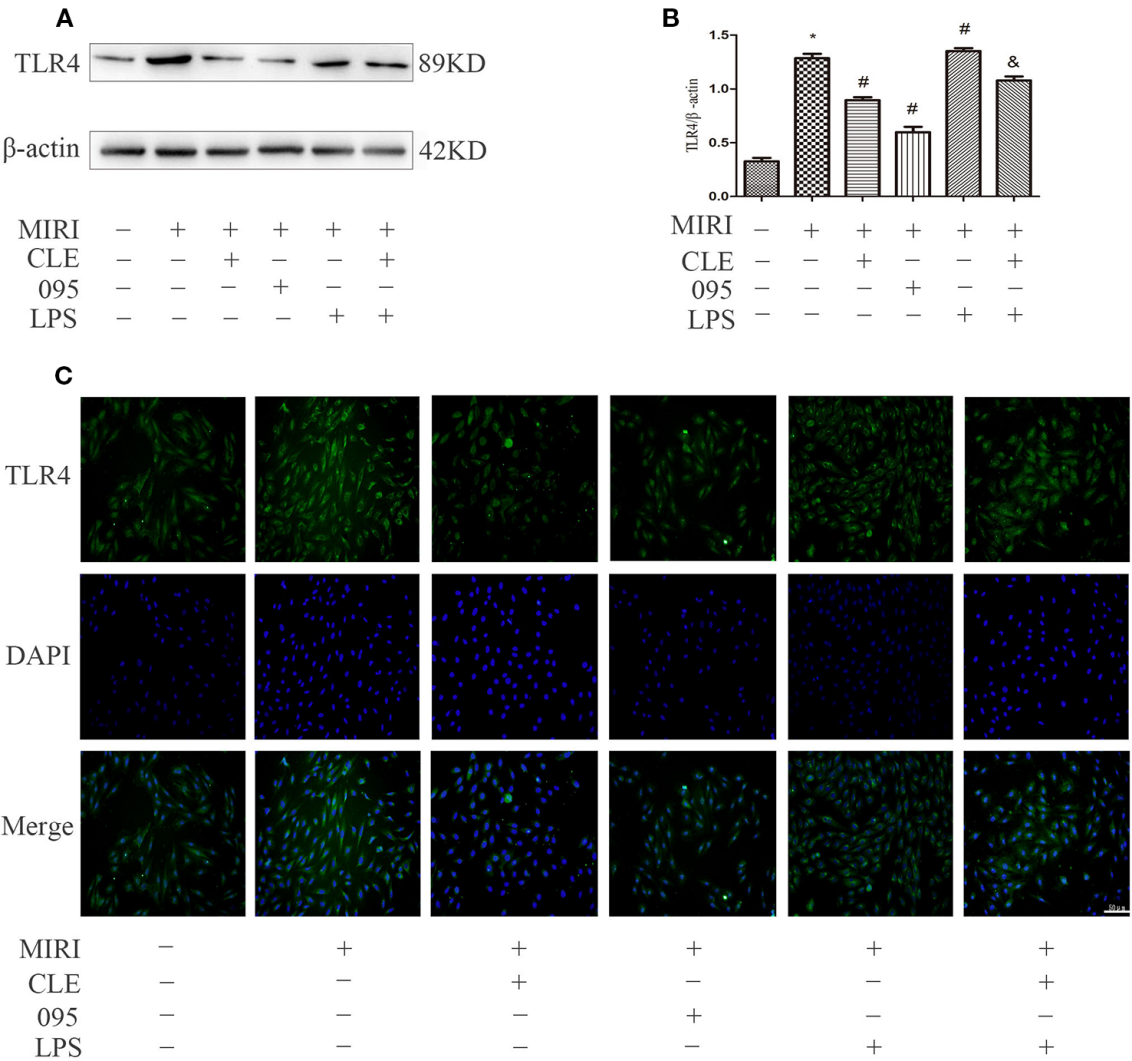
Comparison of TLR4 expressions in H9C2 cardiomyocytes among groups using Western blotting and immunofluorescence. **(A)** Bar graphs showing TLR4 expressions in H9C2 cardiomyocytes of each group. **(B)** Statistical analysis graph of each group indicating TLR4 levels in H9C2 cardiomyocytes; error bars represent SD (standard deviation), *indicates a significant difference compared with CON group, *P* < 0.05; ^#^indicates a significant difference compared with myocardial ischemia-reperfusion injury (MIRI) group, *P* < 0.05; ^&^indicates a significant difference compared with MIRI+LPS group, *P* < 0.05. **(C)** Immunofluorescence staining showing TLR4 expressions in H9C2 cardiomyocytes of each group. Green represents TLR4 and blue represents the nuclei. Scale bar: 50 μm.

In myocardial ischemia-reperfusion injury, the expression of inflammatory protein TLR4 and molecules in the PI3K/Akt signaling pathway was upregulated. To explore the relationship between the mechanism of CLE in myocardial protection and the TLR4/PI3K/Akt signaling pathway, protein expression was observed using Western blotting and immunofluorescence. To further verify the protective effect of CLE on myocardial ischemia-reperfusion injury, we added CMs and HL-1 cells. As shown in **Figures 6A, B**, TLR4 expression level was low in H9C2 cardiomyocytes of the control group. The MIRI group had a significantly higher level of TLR4 expression than the control group; compared with MIRI, TLR4 was significantly down-regulated in the MIRI+CLE group and the MIRI+095 group; compared with the MIRI+LPS group, TLR4 expression level decreased in the MIRI+LPS+CLE group (*P* < 0.05). The fluorescence intensity of TLR4 reflected a similar trend with protein levels (**Figure 6C**). **Supplemental Figures 3A, B** shows that TLR4 expression level was low in CMs of the control group. The MIRI group had a significantly higher level of TLR4 expression than the control group; compared with MIRI, TLR4 was significantly down-regulated in the MIRI+CLE group and the MIRI+095 group; compared with the MIRI+LPS group, TLR4 expression level decreased in the MIRI+LPS+CLE group (*P* < 0.05). **Supplemental Figure 4** shows that the trend of TLR4 expression in each group of HL-1 cells is the same as that of CMs.

**Figure 7:**
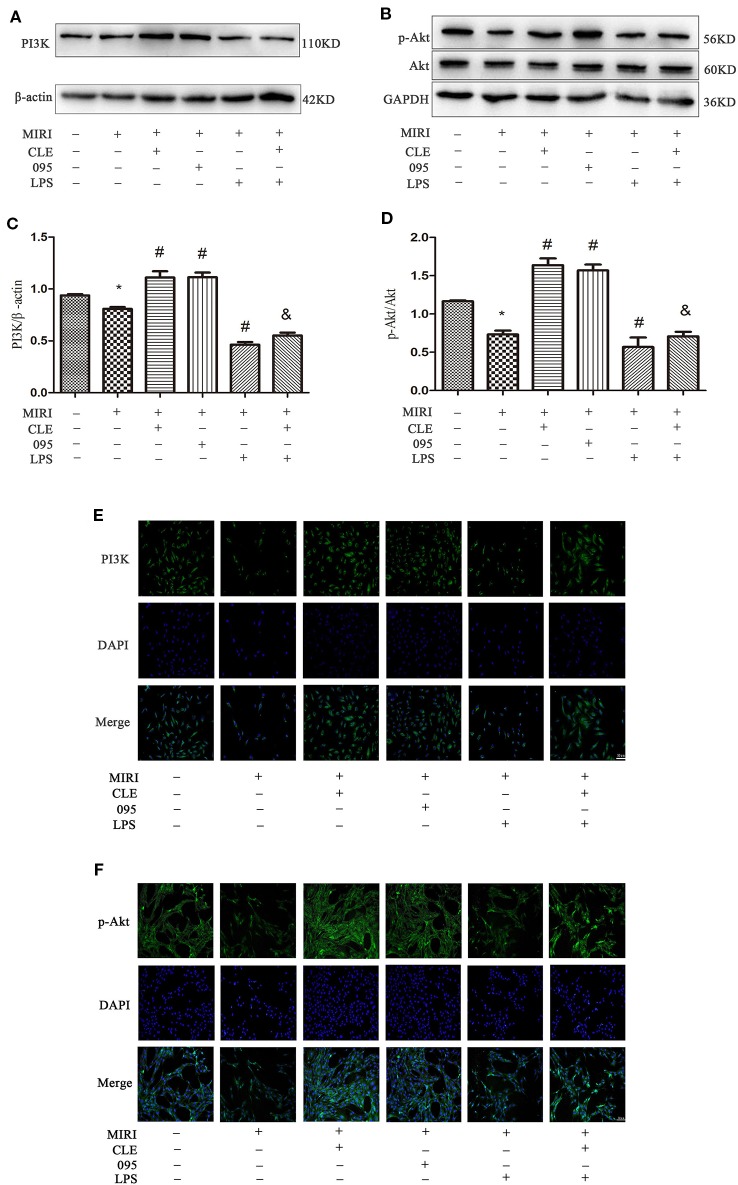
Comparison of PI3K/Akt expressions in H9C2 cardiomyocytes among groups using Western blotting and immunofluorescence. **(A)** Bar graphs showing PI3K expressions in H9C2 cardiomyocytes of each group. **(B)** Bar graphs showing p-Akt and Akt expressions in H9C2 cardiomyocytes of each group. **(C)** Statistical analysis graph of each group indicating of PI3K levels in H9C2 cardiomyocytes. **(D)** Statistical analysis graph of each group indicating of p-Akt/Akt levels in H9C2 cardiomyocytes; Error bars represent SD (standard deviation), *indicates a significant difference compared with CON group, *P* < 0.05; ^#^indicates a significant difference compared with myocardial ischemia-reperfusion injury (MIRI) group, *P* < 0.05; ^&^indicates a significant difference compared with MIRI+lipopolysaccharide (LPS) group, *P* < 0.05. **(E)** Immunofluorescence staining showing PI3K expressions in H9C2 cardiomyocytes of each group. Green represents PI3K and blue represents the nuclei. Scale bar: 50 μm. **(F)** Immunofluorescence staining showing p-Akt expressions in H9C2 cardiomyocytes of each group. Green represents p-Akt and blue represents the nuclei. Scale bar: 50 μm.

Protein detection was performed on the downstream PI3K/Akt signaling pathway, and the results were shown in **Figures 7A–D**. PI3K and p-Akt expression in the MIRI group was significantly lower than that in the control group; PI3K and p-Akt was significantly enhanced in MIRI+CLE and MIRI+095 groups compared with MIRI; compared with MIRI+LPS group, PI3K, and p-Akt expression levels were increased in the MIRI+LPS+CLE group (both *P* < 0.05). The fluorescence intensity of PI3K and p-Akt reflected a similar trend with protein levels (**Figures 7E, F**). From the **Supplemental Figures 3C–F**, we can learn that PI3K and p-Akt expression in CMs the MIRI group was significantly lower than that in the control group; PI3K and p-Akt was significantly enhanced in MIRI+CLE and MIRI+095 groups compared with MIRI; compared with MIRI+LPS group, PI3K, and p-Akt expression levels were increased in the MIRI+LPS+CLE group (both *P* < 0.05).

The results showed that CLE can reduce the inflammatory response induced by myocardial ischemia-reperfusion injury by down-regulating the expression of TLR4. Inhibition or stimulation of TLR4 expression also caused changes in the downstream PI3K/Akt signaling pathway. TLR4 and PI3K/Akt were negatively correlated. This suggested that CLE exerted a protective effect on myocardial ischemia-reperfusion injury through the TLR4/PI3K/Akt signaling pathway.

### Effects of Clemastine Fumarate, CLI-095, and Lipopolysaccharide Treatment on H9C2 Cardiomyocytes Apoptosis

**Figure 8:**
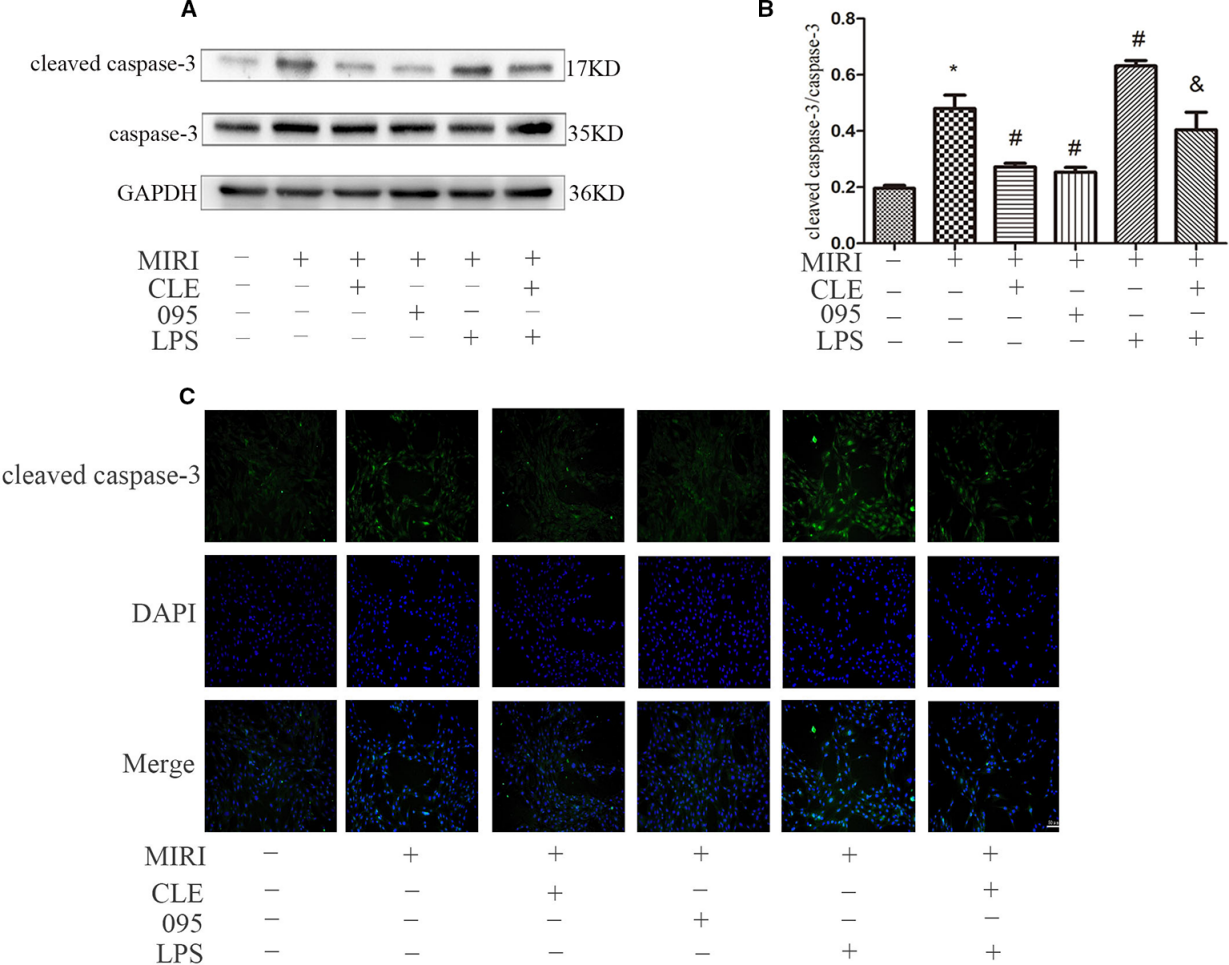
Comparison of cleaved caspase-3 expressions in H9C2 cardiomyocyte among groups using Western blotting and immunofluorescence. **(A)** Bar graphs showing cleaved caspase-3 and caspase-3 expressions in H9C2 cardiomyocytes of each group. **(B)** Statistical analysis graph of each group indicating cleaved caspase-3/caspase-3 levels in H9C2 cardiomyocyte; error bars represent SD (standard deviation), *indicates a significant difference compared with CON group, *P* < 0.05; ^#^indicates a significant difference compared with myocardial ischemia-reperfusion injury (MIRI) group, *P* < 0.05; ^&^indicates a significant difference compared with MIRI+LPS group, *P* < 0.05. **(C)** Immunofluorescence staining showing cleaved caspase-3 expressions in H9C2 cardiomyocyte of each group. Green represents cleaved caspase-3 and blue represents the nuclei. Scale bar: 50 μm.

As cleaved caspase-3 was thought to play major roles in the determination of cell survival or death after apoptotic stimuli, their expression levels in H9C2 cells were determined in order to determine the mechanism by which CLE decreases apoptosis. As shown in **Figures 8A, B**, cleaved caspase-3 expression was low in H9C2 cardiomyocytes of the control group. MIRI group had significantly higher level of cleaved caspase-3 expression than that in the control group; compared with MIRI, cleaved caspase-3 was significantly down-regulated in MIRI+CLE group and MIRI+095 groups; compared with the MIRI+LPS group, cleaved caspase-3 expression level decreased in the MIRI+LPS+CLE group (both *P* < 0.05). The fluorescence intensity of cleaved caspase-3 reflected a similar trend with protein levels (**Figure 8C**). The results showed that CLE could inhibit the production of pro-apoptotic proteins and reduce myocardial apoptosis induced by myocardial ischemia-reperfusion injury. The TLR4 inhibitor CLI-095 had the same effect, while the TLR4 agonist LPS had the opposite effect. CLE could weaken the damage to the myocardium of LPS.

## Discussion

It has been shown that CLE has a protective effect on H9C2 cardiomyocytes and this effect might be associated with TLR4 (Yan et al., 2019). However, the specific mechanism of how CLE affects MIRI remains unclear and the mechanism underlying this protective effect is worth exploring. In this study, we explored the effects of CLE on the regulation of MIRI and the underlying molecular mechanism related to the TLR4/PI3K/Akt signaling pathway in cardiomyocytes and found that CLE could activate the TLR4/PI3K/Akt signaling pathway to relieve MIRI. Importantly, TLR4 signaling was proven to play a critical role in this process. Furthermore, the present study was carried out to elucidate the underlying mechanisms of CLE’s cardioprotective action through the attenuation or enhancement of TLR4 signaling. This study could help understanding of the pharmacology of CLE in the treatment of ischemic heart disease.

Ischemia-reperfusion occurring in cardiomyocytes induces an inflammatory cascade, leading to further damage of the myocardium. Studies have found that the mechanism of MIRI may be related to inflammatory reactions, releasing oxygen free radicals, energy metabolic disorders, apoptosis, calcium overloading (Xiao et al., 2017). Therefore, it is extremely important to delve into the mechanism of pathogenesis of MIRI and find out the prevention and treatment measures.

Clemastine fumarate belongs to the second generation of H1 receptor blockers. Studies have shown that clemastine fumarate not only has antihistamine effects, but also inhibits NF-κB activity (Leurs et al., 2002) and down-regulates TLR4 expression (Eppinger et al., 1995), thereby reducing the synthesis of pro-inflammatory factors and exerting anti-inflammatory effects. Results in our previous study have demonstrated that CLE has a protective effect on MIRI of H9C2 cardiomyocytes and that this effect may be associated with TLR4 (Yan et al., 2019). However, the protective mechanism of CLE in H9C2 cardiomyocytes MIRI has not been elucidated. In this study, we demonstrated that CLE can attenuate apoptosis by inhibiting inflammatory responses; in addition, our results showed that CLE inhibited inflammatory responses and protected cardiomyocytes through the TLR4/PI3K/Akt signaling pathway.

In order to confirm that CLE plays a cardioprotective role and that the downstream PI3K/Akt signaling pathway is activated by TLR4, the TLR4 inhibitor CLI-095 and activator LPS were used in our study. We investigated the effect of different doses of CLI-095 and LPS on H9C2 cardiomyocytes exposed to MIRI. The results showed that CLI-095 had the greatest effect at 1.0 μg/ml and LPS at 5.0 μg/ml.

Increased LDH activity in culture medium is an important indicator of the degree of myocardial ischemia (Ruiz-Gines et al., 2000).The results of this experiment showed that when H9C2 cardiomyocytes were exposed to MIRI, LDH release increased. CLE intervention reduced MIRI cardiomyocyte injury and LDH release reduced. CLI-095 reduced LDH release. LPS increased LDH release and aggravated cell damage, but this effect can be reversed by CLE. These results demonstrated that CLE exerted certain anti-injury effect by decreasing TLR4 expression.

During ischemia, the production of oxygen free radicals increases, and excessive free radicals in the cells can destroy the structure of mitochondria. With the mitochondria swollen and the membrane fluidity being reduced, the oxidative phosphorylation function of the mitochondria is impaired. At the same time, the ATP production is reduced, the energy-dependent calcium pump function of the cell membrane, sarcoplasmic reticulum, and endoplasmic reticulum membrane are lost, resulting in increased intracellular calcium ions and intracellular calcium overload (Ansari and Kurian, 2019; Ke et al., 2019). In addition, during reperfusion, cell membrane lipid peroxidation is enhanced, and cell structure is destroyed. Calcium ions flow in large amounts along the chemical gradient, which results in calcium overload and further aggravates myocardial damage (Vassalle and Lin, 2004). The results of this study showed that although the intracellular calcium ion fluorescence intensity increased during MIRI, but CLE alleviated the calcium disorder caused by MIRI, and reduced the fluorescence intensity of calcium ions. CLI-095 weakened intracellular calcium ion fluorescence intensity, and LPS enhanced it. The results demonstrated that CLE enhances calcium regulation and reduces cardiomyocyte injury by down-regulating TLR4 expression.

When myocardial ischemia-reperfusion injury occurs, the expression of pro-inflammatory factors increased, mainly due to the increased release of TNF-α and IL-1β (Qiu et al., 2019; Xu et al., 2019). CLE can inhibit the release of inflammatory factors such as TNF-α and IL-1β, revers the inflammatory response of MIRI. The release of inflammatory factors decreased after the addition of TLR4 inhibitor CLI-095. TLR4 agonist LPS promoted the release of TNF-α and IL-1β. This effect was reversed by CLE, suggesting that CLE exerts certain anti-inflammatory effect by down-regulating TLR4 expression.

When tissue cells are hypoxic, oxidative stress occurred because of excessive production of reactive oxygen species or insufficient antioxidant capacity of the body. SOD is one of the important substances reflecting the body’s antioxidant activity, which protects the body tissues from damage. The activity of SOD indirectly reflects the body’s ability to scavenge free radicals and resist oxidative damage (Liu et al., 2009). However, oxygen free radicals can be further decomposed to form MDA, which further damages myocardial cells and aggravates myocardial ischemia, allowing MDA content to reflect the degree of cell damage (Tunc et al., 2009). In our study, we found that the antioxidative capacity of cardiomyocytes weakened and cell damage aggravated when H9C2 cardiomyocytes were in MIRI, indicating by the decreased SOD activity and increased MDA content. CLE increased SOD activity and decreased MDA content by enhancing the antioxidant capacity of cells and reducing cell damage. After adding TLR4 inhibitor CLI-095, the antioxidant capacity of cells increased, and cell damage was alleviated. After adding TLR4 agonist LPS, the antioxidant capacity attenuated and this effect was reversed by CLE. These data demonstrated that CLE exerts antioxidant effects by reducing TLR4 expression.

Numerous studies have shown that the PI3K/Akt pathway plays an important role in myocardial protection in MIRI. Studies have found that TLR4 plays an important regulatory role in MIRI and that blocking the TLR4 signaling pathway can reduce the inflammatory response in the MIRI model and reduce myocardial apoptosis (van der Kaaij and Bogers, 2008; Yang et al., 2015). Related studies have shown that stimulation of TLR4 can regulate the PI3K/Akt pathway through the adaptor protein RIP (Rodriguez and Green, 2018). In the ischemic heart model, the expression of the PI3K/Akt pathway is increased by using an immunoregulator to attenuate the expression of the TLR4 signaling pathway. These findings indicate that TLR4 is an upstream factor of the PI3K/Akt signaling pathway, and that TLR4 is negatively correlated with the PI3K/Akt pathway (Ha et al., 2011). In order to investigate whether the protective mechanism of CLE on myocardial ischemia-reperfusion injury is related to TLR4-mediated TLR4/PI3K/Akt signaling, the expression of pathway-related proteins was detected by Western blotting and immunofluorescence. The results showed that CLE could attenuate the expression of TLR4, cleaved caspase-3/caspase-3 protein in MIRI cells and attenuate the expression of corresponding proteins. CLE enhanced the expression of PI3K and p-Akt/Akt. However, TLR4 inhibitor CLI-095 inhibited the TLR4 signal and promoted expression of PI3K and p-Akt/Akt. The TLR4 signal was stimulated by the TLR4 agonist LPS, but the PI3K and p-Akt/Akt proteins expression were inhibited. Therefore, the protective effect of CLE in MIRI was mainly achieved by regulating TLR4/PI3K/Akt, and TLR4 played a key role in this mechanism.

Through a series of experiments on H9C2 cells, we verified that CLE can suppress myocardial ischemia-reperfusion injury by stimulating TLR4 and downstream PI3K/Akt signaling pathways to protect myocardial cells. To further validate the effect of CLE on the MIRI primary cardiomyocytes and HL-1 cells, we detected the expression levels of inflammatory factors TNF-α and IL-1β on the mRNA level by PCR technology and expression of related pathway proteins with Western blot technology. It can be seen from the results that not only in H9C2 cells, but also in CMs and HL-1 cells, CLE reduced the inflammatory response of myocardial ischemia-reperfusion injury through the regulation of TLR4.

TLR4 is expressed in the heart and up-regulated during myocardial ischemia/reperfusion (I/R) injury. Stimulation of TLR4 leads to the activation of intracellular signaling pathways, e.g., activation of NF-κB, and results in the production of pro-inflammatory cytokines, such as TNF-α and IL-1β, leading to heart damage and dysfunction. Stimulation of TLR4 can regulate the PI3K/Akt pathway through the adaptor protein RIP. TLR4 stimulation leads to tyrosine phosphorylation of the toll-interleukin receptor (TIR) domain. The TIR domain subsequently disassociates from MyD88, binds the p85 regulatory subunit of PI3K, and phosphorylates Akt. Afterwards, activated Akt promotes survival and inhibits the apoptosis of cardiac myocytes.

H1 receptor activation can accelerate the generation of oxygen free radicals, induce neutrophil aggregation, and trigger an inflammatory response. CLE belongs to the second-generation H1 receptor blocker. It not only has antihistamine effect, but also inhibits NF-κB activity (Leurs et al., 2002) and down-regulates TLR4 expression. TLR4 is involved in the immune response to exogenous microbial infection. The first line of defense is the key upstream factor that activates the inflammatory response (Sun et al., 2015).

In conclusion, we demonstrated that CLE can activate the PI3K/Akt signaling pathway through the TLR4 receptor, affect the inflammatory response, oxidative stress, cell damage, and apoptosis in cardiomyocytes, and alleviate myocardial ischemia-reperfusion injury.

## Data Availability Statement

The datasets generated for this study are available on request to the corresponding authors.

## Author Contributions

ZJ, RZ, and XS developed the concept and design of the study. XY performed the experiments and wrote the first draft. XY, RY, FY, YH, JY, and XX performed the experiments and analyzed the results. ZJ, RZ, and XS analyzed the data and edited the manuscript.

## Funding

This study was supported by the Natural Science Foundation of Shandong Province (ZR2017LH002 and ZR2017MH066).

## Conflict of Interest

The authors declare that the research was conducted in the absence of any commercial or financial relationships that could be construed as a potential conflict of interest.
