# Supplementary material for: Clemastine Fumarate Protects Against Myocardial Ischemia Reperfusion Injury by Activating the TLR4/PI3K/Akt Signaling Pathway
Source: Front Pharmacol. 2020 Feb 10;11:28. doi: 10.3389/fphar.2020.00028 (PMC7025565; doi:10.3389/fphar.2020.00028)
Supplement: Supplementary file 1 [file DataSheet_1.docx]

Supplementary Material

**Clemastine Fumarate Protects Against Myocardial Ischemia Reperfusion Injury by Activating the TLR4/PI3K/Akt Signaling Pathway**

**Supplementary Data**

**SUPPLEMENTARY FIGURE LEGENDS:**

**FIGURE S1 |**  **Comparison of the expression of TNF-αand IL-1βmRNA in CMs by qPCR. (A)** Statistical analysis graph of each group indicating the expression of TNF-α in CMs. **(B)** Statistical analysis graph of each group indicating the expression of IL-1β in CMs. Error bars represent SD (standard deviation), * indicates a significant difference compared with CON group, *P* < 0.05; # indicates a significant difference compared with MIRI group, *P* < 0.05; & indicates a significant difference compared with MIRI+LPS group, *P* < 0.05.

**FIGURE S2 |**  **Comparison of the expression of TNF-αand IL-1βmRNA in HL-1 cells by qPCR. (A)** Statistical analysis graph of each group indicating the expression of TNF-α in HL-1 cells. (B) Statistical analysis graph of each group indicating the expression of IL-1β in HL-1 cells. Error bars represent SD (standard deviation), * indicates a significant difference compared with CON group, *P* < 0.05; # indicates a significant difference compared with MIRI group, *P* < 0.05; & indicates a significant difference compared with MIRI+LPS group, *P* < 0.05.

**FIGURE S3 |** Comparison of TLR4、PI3K/Akt expressions in CMs among groups using Western blotting and immunofluorescence. **(A)** Bar graphs showing TLR4 expressions in CMs of each group. **(B)** Statistical analysis graph of each group indicating TLR4 levels in CMs ; Error bars represent SD (standard deviation), * indicates a significant difference compared with CON group, *P* < 0.05; # indicates a significant difference compared with MIRI group, *P* < 0.05; & indicates a significant difference compared with MIRI+LPS group, *P* < 0.05. **(C)** Bar graphs showing PI3K expressions in CMs of each group. **(D)** Bar graphs showing p-Akt and Akt expressions in CMs of each group. **(E)** Statistical analysis graph of each group indicating of PI3K levels in CMs. **(F)** Statistical analysis graph of each group indicating of p-Akt/Akt levels in CMs; Error bars represent SD (standard deviation), * indicates a significant difference compared with CON group, *P* < 0.05; # indicates a significant difference compared with MIRI group, *P* < 0.05; & indicates a significant difference compared with MIRI+LPS group, *P* < 0.05.

**FIGURE S4 |** Comparison of TLR4 expressions in HL-1cells among groups using Western blotting and immunofluorescence. **(A)** Bar graphs showing TLR4 expressions in HL-1cells of each group. **(B)** Statistical analysis graph of each group indicating TLR4 levels in HL-1cells ; Error bars represent SD (standard deviation), * indicates a significant difference compared with CON group, *P* < 0.05; # indicates a significant difference compared with MIRI group, *P* < 0.05; & indicates a significant difference compared with MIRI+LPS group, *P* < 0.05.

**Supplemental results**

Our results showed that MIRI aggravates the inflammatory response of CMs, and HL-1 cells, increasing the mRNA expression levels of TNF-α and IL-1β. Meanwhile, MIRI can upregulate the expression of TLR4 protein in CMs, and HL-1 cells and down-regulated the expression of PI3K/Akt protein in CMs, CFs. After treatment with CLE, the expressions of TNF-α mRNA and IL-1β mRNA were declined. TLR4 protein expression of CMs, and HL-1 cells treated with CLE declined, but PI3K/Akt protein expression increased in CMs. In addition, after the action of the TLR4 inhibitor CLI-095, the results were similar to those of CLE. Meanwhile, using of TLR4 agonist LPS aggravated the related reactions caused by MIRI. The role of LPS was reversed after using CLE. These results suggested that CLE could activate the PI3K/Akt signaling pathway by acting on the TLR4 receptor, thereby attenuating MIRI in CMs. In addition, CLE could down-regulate TLR4 expression and reduce the inflammatory response caused by MIRI in HL-1 cells. In conclusion, this study provided evidence that CLE could activate the TLR4/PI3K/Akt signaling pathway to relieve MIRI in CMs, and that TLR4 played a key role in this pathway. CLE could affect the inflammatory response of MIRI in HL-1 cells by regulating TLR4.
