# Supplementary figures and images for: Clemastine Fumarate Protects Against Myocardial Ischemia Reperfusion Injury by Activating the TLR4/PI3K/Akt Signaling Pathway
Source: Front Pharmacol. 2020 Feb 10;11:28. doi: 10.3389/fphar.2020.00028 (PMC7025565; doi:10.3389/fphar.2020.00028)

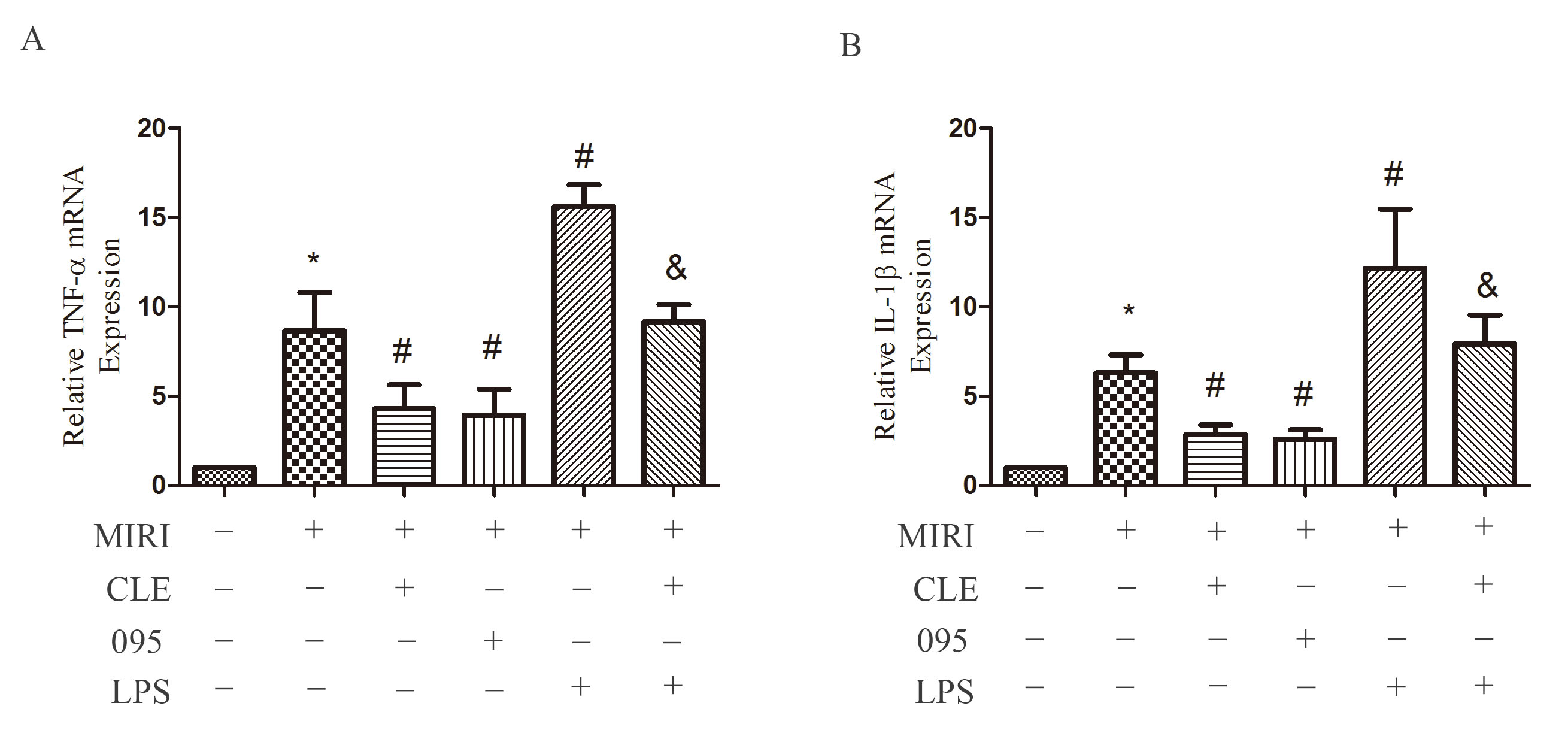

Supplement: Supplementary file 2 [file Image_1.jpg]

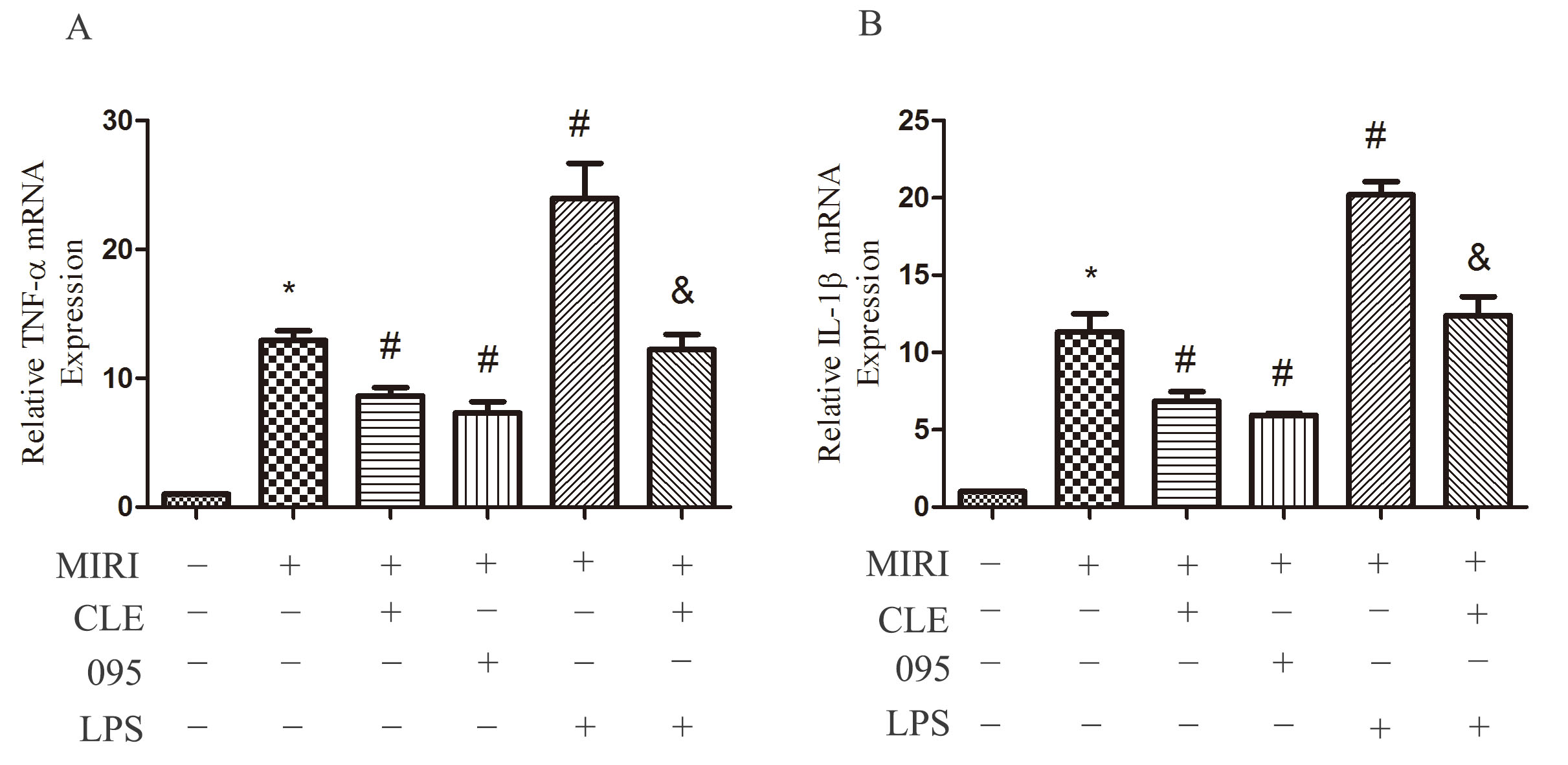

Supplement: Supplementary file 3 [file Image_2.jpg]

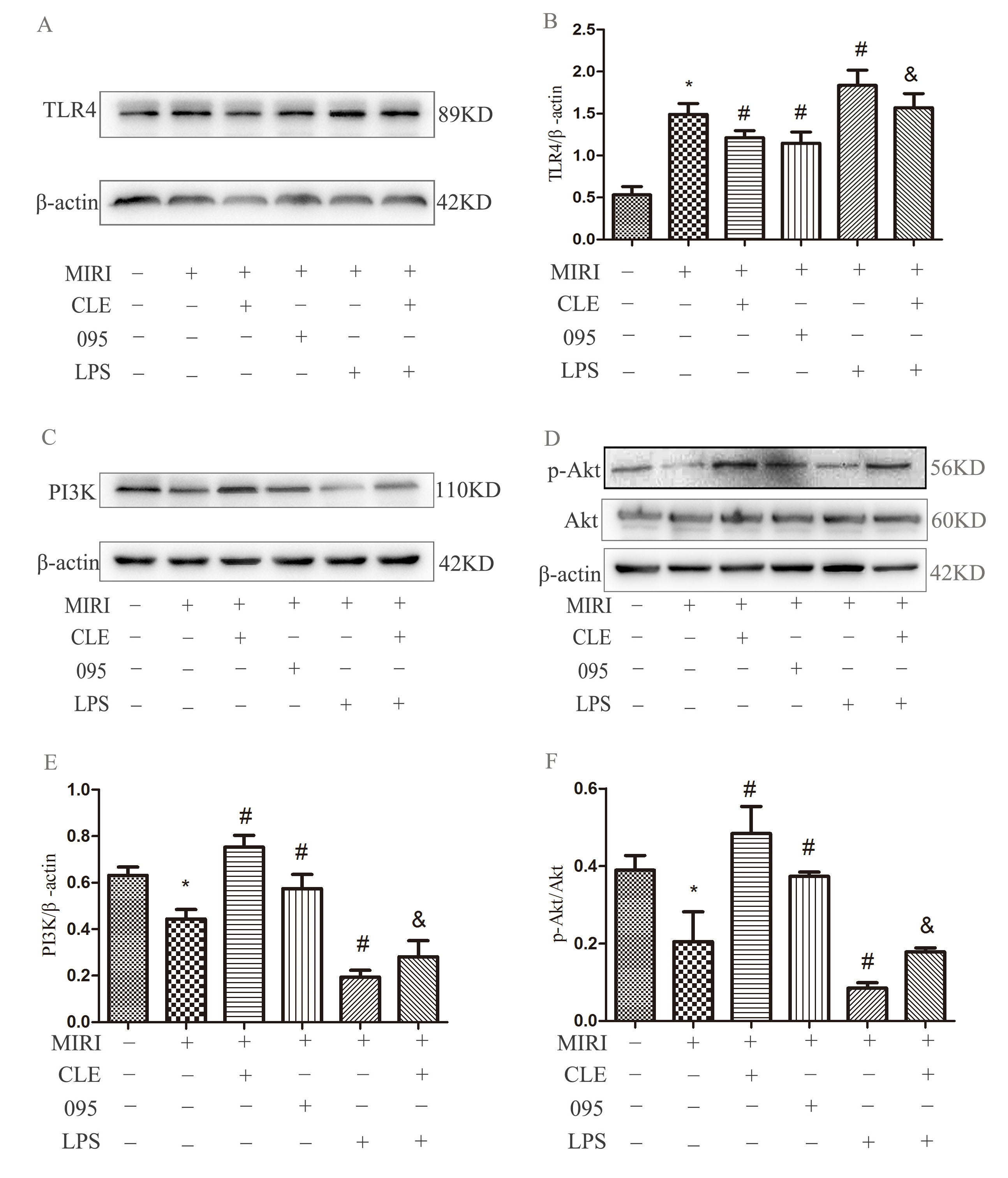

Supplement: Supplementary file 4 [file Image_3.jpg]

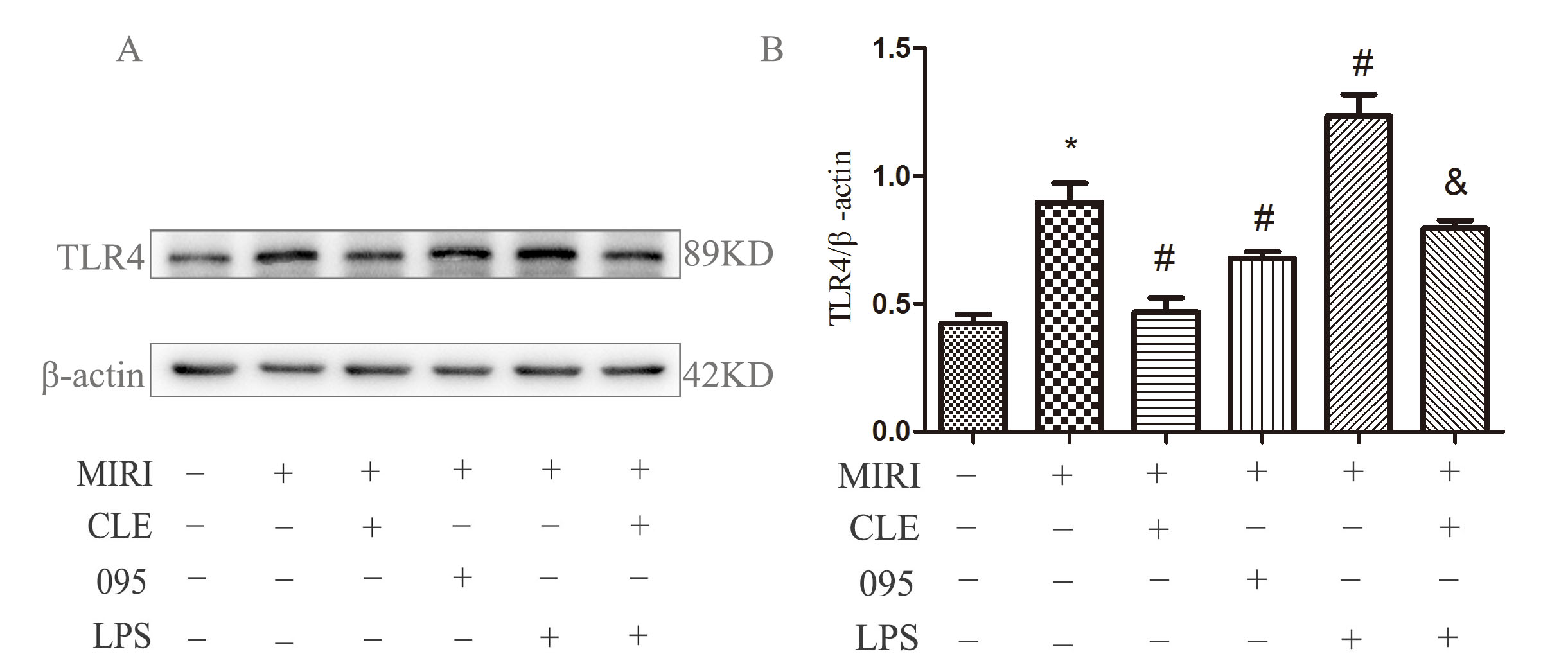

Supplement: Supplementary file 5 [file Image_4.jpg]
